# The alteration of IL-17 signaling pathway in bipolar disorder: a preliminary study with transcriptomic perspective

**DOI:** 10.3389/fpsyt.2025.1539038

**Published:** 2025-06-05

**Authors:** Xiaobo Wang, Su Yu, Zhen Gao, Fangming Xu, Yumei Wang, Tianle Zhang, Tingting Xie, Xihua Jia

**Affiliations:** ^1^ Department of Psychiatry, the First Hospital of Hebei Medical University, Shijiazhuang, Hebei, China; ^2^ Department of Clinical Psychology, The First Central Hospital of Baoding, Baoding, Hebei, China; ^3^ Institute of Brain Science and Brain-Inspired Research, Shandong First Medical University & Shandong Academy of Medical Sciences, Jinan, Shangdong, China; ^4^ Departmentof Psychology, Shandong Provincial Hospital Affiliated to Shandong First Medical University, Jinan, Shangdong, China; ^5^ Department of Oncology, The First Central Hospital of Baoding, Baoding, Hebei, China

**Keywords:** bipolar disorder, DEGs (differentially expressed genes), IL-17 pathway, immunoinflammatory response, immune infiltration analysis

## Abstract

**Objective:**

Bipolar Disorder (BD) is a complex psychiatric condition influenced by genetic and environmental factors. This study aims to explore global mRNA expression in Peripheral blood white cells (PBMC) of BD patients.

**Methods:**

We analyzed whole-genome gene expression profiles from PBMC of 12 BD states-manic (BD-M), 12 BD states-depressive (BD-D), and 12 matched healthy controls (HC) using microarrays. Validation of differentially expressed genes within the IL-17 pathway was conducted through qRT-PCR, while ELISA measured specific protein levels. GO and KEGG pathway enrichment analyses were performed, alongside PPI analysis and Gene Set Variation Analysis (GSVA) to assess immune cell infiltration.

**Results:**

In the comparison between BD and HC, a total of 304 differentially expressed genes (DEGs) were identified, of which 217 genes were upregulated and 87 genes were downregulated. In the comparisons between BD and HC, BD-M and HC, and BD-D and HC, 91 DEGs were found. Enrichment analysis suggested that biological processes and signaling pathways related to extracellular matrix, stress response, heparin binding, and immune inflammation were upregulated in the BD group. KEGG analysis and PPI results indicate that the IL-17 signaling pathway plays an important role in BD and its subtypes. In the IL-17 signaling pathway, compared to HC, the expression of *Jun, Fosb, Fosl1*, *TNFAIP3*, *NFKBIA*, *CXCL2*, *CXCL8*, *IL6* and *IL17* genes was upregulated in BD patients. RT-qPCR analysis showed that the expression of J*un*, *Fosb*, *Fosl1*, *NFKBIA*, *TNFAIP3*, *CXCL2*, and *CXCL8* genes was significantly upregulated in BD, ELISA results indicated that the protein levels of CXCL8/IL-8, IL-6 and IL-17 in the BD group were significantly higher than those in the HC group (all P < 0.05). Immune infiltration analysis showed that central memory CD4+ T cells (P=0.010), eosinophils (P=0.038), and mast cells (P=0.029) were increased in infiltration in the BD group.

**Conclusion:**

This study integrates transcriptomics and immune microenvironment analysis, suggesting that the IL-17 signaling pathway may be involved in the pathogenesis of BD and its subtypes through inflammation triggered by chemokines. This research deepens our understanding of the molecular mechanisms of BD and emphasizes the importance of the immune system in its pathology.

## 1 Introduction

Bipolar Disorder (BD) represents a significant global health challenge, marked by debilitating episodes of depression and mania/hypomania. It affects approximately 2% of the global population and is characterized by high morbidity, mortality, and disability rates (1–3). Notably, BD usually appears around age 20, has a chronic course, and increases suicide risk, making it a major public health concern and a leading cause of disability globally (4, 5). Current research underscores the critical interplay between genetic predispositions and environmental triggers in BD’s onset (6). Twin and family studies estimate BD’s heritability between 60–85%, highlighting a substantial genetic contribution (2, 7, 8). However, only a fraction of the genetic factors are identified. The complexity of BD’s etiology likely stems from a network of multiple, interacting genetic elements, underscoring the need for further elucidation of its biological mechanisms.

In recent years, the immuno-inflammatory hypothesis has been one of the key points of BD exploration, and studies have shown that BD has immune/inflammatory mechanisms accompanied by chronic and low-grade inflammatory states (9). It is suggested that inflammatory markers suggestive of a low-grade inflammatory state may play a role in the neurocognitive impairments associated with BD (10), while the levels of immune inflammation intensify during manic and depressive episodes (11). The neutrophil-to-lymphocyte ratio (NLR) in BD patients has been recognized as a novel inflammatory marker that may correlate with the intensity of depressive symptoms (12). Furthermore, immune analysis shows enhanced anti-inflammatory responses in those on antidepressants, suggesting immune function rejuvenation may improve symptoms (13).There is also emerging evidence that BD Patients have a higher probability of developing autoimmune diseases, while patients with autoimmune disorders are at a relatively high risk for BD (14). For example, the prevalence of BD in patients with systemic lupus erythematosus will be higher compared to the control group (15). These studies suggest that immuno-inflammatory dysfunction may be a critical factor in the disease progression of BD.

Gene expression research using microarray technology provides insights into new genetic opportunities for complex diseases. Traditionally, gene expression studies in BD patients have focused on post-mortem brain tissue, which limits the ability to conduct real-time analyses on living patients (16–18). The gene expression patterns in peripheral blood offer a less invasive and more accessible method suitable for longitudinal studies (19, 20). However, it is important to note that gene expression patterns may be constrained by the spatiotemporal dynamics of inflammation-related pathways; for example, in individuals with depression, inflammatory markers in peripheral blood may differ from the expression patterns in the dorsolateral prefrontal cortex (dlPFC) (21). This suggests that the indicative value of peripheral markers needs further confirmation in the context of chronic neurodegeneration.

We are aware that few studies have focused on the differential gene expression in the peripheral blood of patients with BD. This study aims to utilize microarray technology to identify the differential expression of genes in peripheral blood mononuclear cells between patients with bipolar disorder, their subtypes, and healthy controls. The study will conduct enrichment analyses with Gene Ontology (GO) and Kyoto Encyclopedia of Genes and Genomes (KEGG), as well as protein-protein interaction (PPI) analysis, to identify signaling pathways and key genes associated with bipolar disorder, validating the central genes that may be involved in the pathogenesis of BD. Additionally, through Gene Set Variation Analysis (GSVA), we will analyze how immune cells infiltrate the samples and further explore how different immune cells interact in bipolar disorder. This will explore the immune inflammatory pathways or factors linked to BD patients, offering a theoretical basis for treating immune inflammation in BD. Furthermore, it will provide new insights into the molecular mechanisms and pathophysiology of bipolar disorder, helping us better understand this complex disease.

## 2 Participants and methods

### 2.1 Participant profiles

This study was conducted at the Mental Health Centre of the First Hospital of Hebei Medical University, Hebei Province, China. The study included 24 patients with bipolar disorder—equally divided between bipolar disorder manic (BD-M) and bipolar disorder depressive (BD-D) —and 12 healthy controls matched for gender and age, all recruited from our hospital between January and July 2023.

The inclusion criteria were as follows: 1) aged between 18 and 60 years; 2) diagnosed with BD according to the 10th Revision of the International Statistical Classification of Diseases and Related Health Problems (ICD-10); 3) having not received treatment for at least two weeks, like medication and therapy. The exclusion criteria were as follows: 1) individuals with other mental disorders, such as depression or schizophrenia; 2) individuals with conditions that may affect inflammatory markers, including acute inflammatory illnesses and metabolic syndromes like autoimmune diseases, infections, blood disorders, diabetes, and heart failure; 3) those with a current or historical substance abuse or dependence within the past six months; Healthy subjects included in the study must meet the following criteria: 1) aged between 18 and 60 years; 2) Structured Clinical Interview (SCID) was used to confirm that participants had no mental disorders; 3) free from conditions that could affect inflammatory markers, including acute inflammatory illnesses and metabolic syndromes such as autoimmune diseases, infections, blood disorders, diabetes, and heart failure.

This research received approval from the Institutional Review Board of our hospital (Approval No. 20220908) prior to data collection, and was performed in accordance with the Declaration of Helsinki and its subsequent revisions. Written informed consent was obtained from all participants, ensuring their understanding of the study’s purpose and procedures, all participants were informed that they were free to withdraw from the study at any time, without giving a reason. All data collected were treated with strict confidentiality and anonymity to protect the participants’ privacy.

### 2.2 Blood sample collection

Peripheral blood samples were obtained from each participant through venipuncture in the morning following an overnight fast. Two 5ml blood samples were collected. The first sample was drawn into a vacutainer tube containing EDTA, facilitating the separation of plasma and PBMC (PBMC are single-nucleus cells found in peripheral blood, which include lymphocytes and monocytes). The second sample was collected in a tube featuring a polymeric gel, which allows for the extraction of serum following centrifugation. These samples were then preserved at -80°C for subsequent analysis.

### 2.3 RNA extraction and microarray analysis

Total RNA was isolated from PBMCs of blood samples obtained from both enrolled patients and healthy control, this extraction utilized the TRIzol reagent (Invitrogen, CA, USA). The assessment of the RNA’s concentration, purity, and integrity was conducted using the NanoDrop spectrophotometer (Thermo Scientific, Massachusetts, USA). Subsequent preparation for hybridization involved the ligation of Illumina PE adapter oligonucleotides to the RNA. The library fragments were then size-selected for cDNA fragments ideally between 400–500 bp, employing the AMPure XP system (Beckman Coulter, Beverly, CA, USA) for purification. Enrichment of DNA fragments, now with adaptor molecules attached at both ends, was achieved through a 15-cycle PCR reaction using the Illumina PCR Primer Cocktail. The PCR products underwent purification using the AMPure XP system and were quantified with the Agilent High Sensitivity DNA assay on a Bioanalyzer 2100 system (Agilent, California, USA). Finally, the prepared sequencing library, was sequenced using the NovaSeq 6000 platform (Illumina, California, USA) facilitated by Shanghai Personal Biotechnology Co., Ltd.

### 2.4 Gene expression analysis

The initial dataset, in FASTQ format (referred to as Raw Data), was processed using the fastp software (version 0.22.0) to filter the sequencing data, thus obtaining high-quality sequences (termed Clean Data). The ComBat method was used to model and remove technical batch effects. For quantification of gene expression, HTSeq (version 0.9.1) was employed to calculate Read Count values for each gene. Subsequently, the expression data were normalized using the Fragments Per Kilobase of transcript per Million mapped reads (FPKM) method. Differential gene expression analysis was conducted utilizing the edge R package (version 3.40.2), with the selection criteria set as follows: an absolute log2 fold change (|log2FoldChange|) greater than 1 and a significant P-value of less than 0.05. Heatmaps were generated to illustrate the expression levels of identical genes across different samples and the varied gene expression patterns within the same sample. This was achieved using the Euclidean method for distance calculation and the Complete Linkage method for clustering. GO terms were assigned to all analyzed genes using the Gene Ontology database, and the top 20 enriched GO terms for each group were identified through GO enrichment analysis performed with the topGO software (version 2.50.0). Additionally, ClusterProfiler software (version 4.6.0) facilitated the enrichment analysis of differential gene expression within the KEGG pathway, concentrating on pathways significantly enriched (P-value < 0.05). A protein-protein interaction network was generated using the STRING database (v12.0) to elucidate the relationships between target genes, providing insights into their potential functional associations. Interactions were filtered with a confidence score threshold of ≥0.7, retaining associations with at least 70% functional relevance, and the generated network was limited to direct interactions between seed proteins (i.e., differentially expressed genes) and their main interactors (first layer). All p-values were adjusted using the Benjamini-Hochberg false discovery rate (FDR) at α=0.05. The above methods compared the overall differences between the BD group and HC, as well as separately between BD-M and HC, BD-D and HC.

### 2.5 Quantitative Real-Time Reverse Transcription PCR

To validate the differential expression of mRNA, qRT-PCR was conducted on a subset of participants, comprising 30 individuals: 10 patients with BD-M) 10 patients with BD-D and 10 healthy controls. Seven key genes in the IL-17 signaling pathway were validated by qRT-PCR, and the expression differences between BD and HC were compared. The primer sequences used are detailed in **Table 1**.

**Table 1:** qPCR primers used in the study.

Gene	Primer—F (5’to3’)	Primer—R (3’to5’)
*Jun*	GAAACGACCTTCTATGAC	GGTCCACTCTCAATCACT
*NFKBIA*	TACACTTAGCCTCTATCCAT	CTCCTGAGCATTGACATC
*Fosb*	ACACAGTGAAGTTCAAGTC	GTGAGGACAAACGAAGAA
*TNFAIP3*	AATGAGATGAAGGAGAAG	ATTGATGAGATGAGTTGT
*Fosl1*	TCCCTAACTCCTTTCACC	CTGCTACTCTTGCGATGA
*CXCL2*	AACATCCAAAGTGTGAAG	ATTCTTGAGTGTGGCTAT
*CXCL8*	TGCTAAAGAACTTAGATGTCAG	GGTCCACTCTCAATCACT
actin	CGAGAAGATGACCCAGAT	GATAGCACAGCCTGGATA

For this analysis, 1 μg of total RNA from each sample was reverse transcribed using the TRIzol reagent (Invitrogen, CA, USA), adhering to the manufacturer’s guidelines. The resulting cDNA was synthesized employing the PrimeScript™ 1st Strand cDNA Synthesis Kit, and PCR amplification was carried out on the EDC-810 PCR machine from Beijing Dongsheng Innovation Biotechnology Co., Ltd. (Beijing, China). The qRT-PCRs were executed using the primers specified in **Table 1** and the AceQ^®^ qPCR SYBR^®^ Green Master Mix on a LightCycler 480 II 384-channel real-time PCR system (Roche, California, USA). The thermal cycling conditions were set as follows: an initial denaturation at 95°C for 5 minutes, followed by 40 cycles of denaturation at 95°C for 15 seconds, annealing at 60°C for 30 seconds, and a standard dissociation protocol to confirm the specificity of each PCR product. Expression levels were normalized against ACTB (β-actin) as the reference gene.

### 2.6 ELISA validation

Peripheral venous blood samples collected from all participants were analyzed using enzyme-linked immunosorbent assay (ELISA) to detect four key cytokines (CXCL2, CXCL8/IL-8, IL-6, and IL-17) in the IL-7 signaling pathway, and the differential expression between BD and HC was compared. The assays utilized ELISA kits supplied by Shanghai Yuanmu Biotechnology Co., Ltd (Shanghai, China), following the procedures outlined by the manufacturer. All kit performance: the linear regression of the standard correlates with the expected concentration correlation coefficient R, which is greater than or equal to 0.9900. The sensitivity of this assay is 1.0 pg/ml.

### 2.7 Evaluation of immune cell infiltration using GSVA

The infiltration levels of 28 distinct immune cell types within the samples from the normal control group and the BD group (comparing 12 healthy controls versus 24 BD patients) were assessed through the single-sample Gene Set Variation Analysis (ssGSEA) algorithm, implemented in the “GSVA” R package. Initially, the expression profiles of these 28 immune cell types between the BD and HC groups were visualized using the “pheatmap” R package (22). The marker gene list comes from the CellMarker 2.0 database (23).Subsequently, the differences in immune cell infiltration between the BD and HC groups were quantified through a box plot analysis. This analysis was performed using the Wilcoxon test method, facilitated by the “ggplot2” R package, highlighting statistically significant disparities between the two groups. Finally, a heatmap illustrating the correlation between characteristic genes and the differentially infiltrated immune cells was generated.

### 2.8 Statistical analysis

Statistical analyses were conducted using SPSS software (version 27.0, SPSS Inc., Chicago, IL) and R programming language, with significance established at a P-value threshold of 0.05. For continuous variables, data adherence to normal distribution was first assessed. Measurement data conforming to normality were presented as mean ± standard deviation (SD) and analyzed using the t-test. Conversely, for data not meeting normality criteria, the Mann-Whitney U test was employed for intergroup statistical comparisons. Count data were analyzed using the Chi-square test. Furthermore, receiver operating characteristic (ROC) curve analysis was applied to identify optimal cut-off values for continuous variables, facilitating the identification of the most predictive biomarkers for BD. The diagnostic performance of these biomarkers was quantified by calculating the area under the ROC curve (AUC), with values ranging from 0 to 100%, indicating the potential efficacy of these variables in predicting BD presence or severity.

## 3 Results

### 3.1 Characteristics of healthy subjects and BD patients

In total, 12 healthy subjects and 24 patients with BD (12 with BD-M and 12 with BD-D) contributed clinical data and peripheral venous blood samples. The demographic characteristics of healthy participants and BD patients are shown in **Table 2**. Statistical analysis indicates that there are no significant differences between the two groups in terms of age, gender, BMI, and smoking.

**Table 2:** Demographic and clinical information for both BD subjects and healthy controls are summarized.

Characteristics	BD-D (N=12)	BD-M (N=12)	BD (N=24)	HC (N=12)	BD Vs HC, p
Age (years)	30.33 ± 11.69	33,58 ± 10.49	31.96 ± 10.99	30.58 ± 9.77	0.701
Male: Female	5:7	5:7	10:14	5:7	NA
BMI (kg/m^2^)	23.09 ± 1.83	24.11 ± 2.36	23.59 ± 2.13	23.87 ± 2.71	0.78
Duration of illness	4 (3-10.5)	7.17 ± 5.65	6.83 ± 5.18	NA	NA
Smoking	3 (25%)	4 (33.33%)	7 (29.17%)	3 (25%)	0.79	
HAM-D score,	30.16 ± 4,17	5.08 ± 0.78	NA	4.75 ± 1.29	NA
YMRS score,	2 (2-4)	25.08 ± 6.45	NA	2.41 ± 0.99	NA

NA, Not Applicable.

### 3.2 Identification of differentially expressed genes

**Figure 1:**
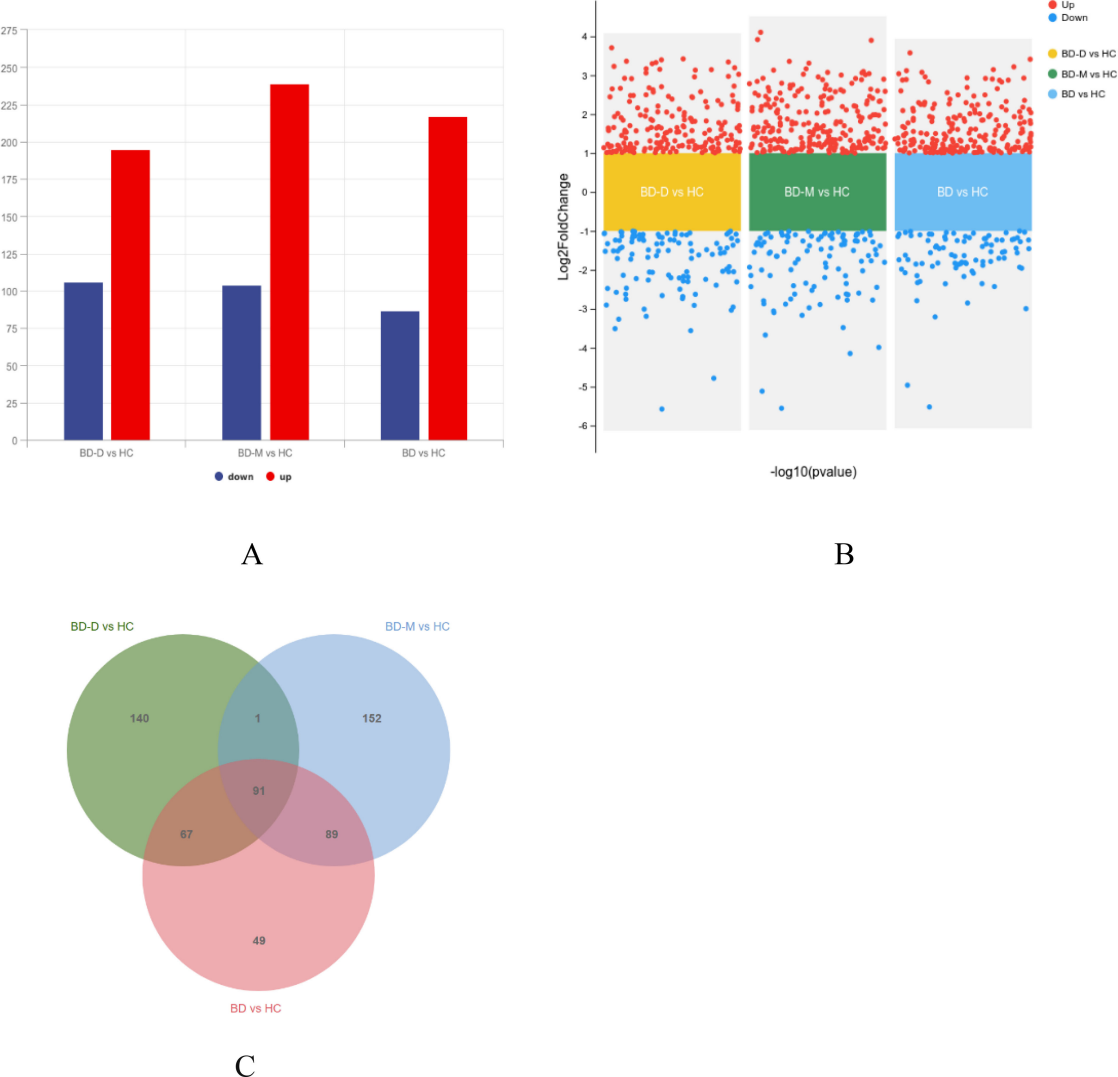
**(A)** Barplot showing Identification of differentiated expressed genes (DEGs) in the BD vs HC, BD-M vs HC, BD-D vs HC group. Red bars indicate the up-regulated genes, while blue represent the down-regulated. **(B)** Volcano plot showing DEGs in the in BD, BD-M, BD-D compared with HC; Red dots indicate the up-regulated genes, while blue represent the down-regulated. **(C)** The Venn diagram compares DEGs from three groups (BD-M vs HC, BD-D vs HC, and BD vs HC).

Through genome-wide microarray analysis of mRNA from PBMC of 36 enrollees, we identified a significant number of DEGs between the groups (BD, BD-D, and BD-M) and HC. Specifically, across the broader BD cohort, 304 DEGs were noted, with 217 genes up-regulated and 87 down-regulated. The BD-M group revealed 343 DEGs, consisting of 239 up-regulated and 104 down-regulated genes. In the BD-D group, 301 DEGs were identified, with 195 genes up-regulated and 106 genes down-regulated. Graphical representations, including bar plots and volcano plots, elucidated the distribution and significance of DEGs between the BD vs. HC, BD-D vs. HC, and BD-M vs. HC groups (**Figures 1A, B**). A Venn diagram highlighted the overlap in DEGs across BD, BD-D, and BD-M, compared with HC, identifying 91 common DEGs (co-DEGs) across these comparisons (**Figure 1C**). A heatmap provided a visual representation of the expression patterns of these 91 co-DEGs in PBMC samples from patients with BD-D, BD-M versus healthy control subjects (**Supplementary Figure S1**).

### 3.3 GO Enrichment analysis

**Figure 2:**
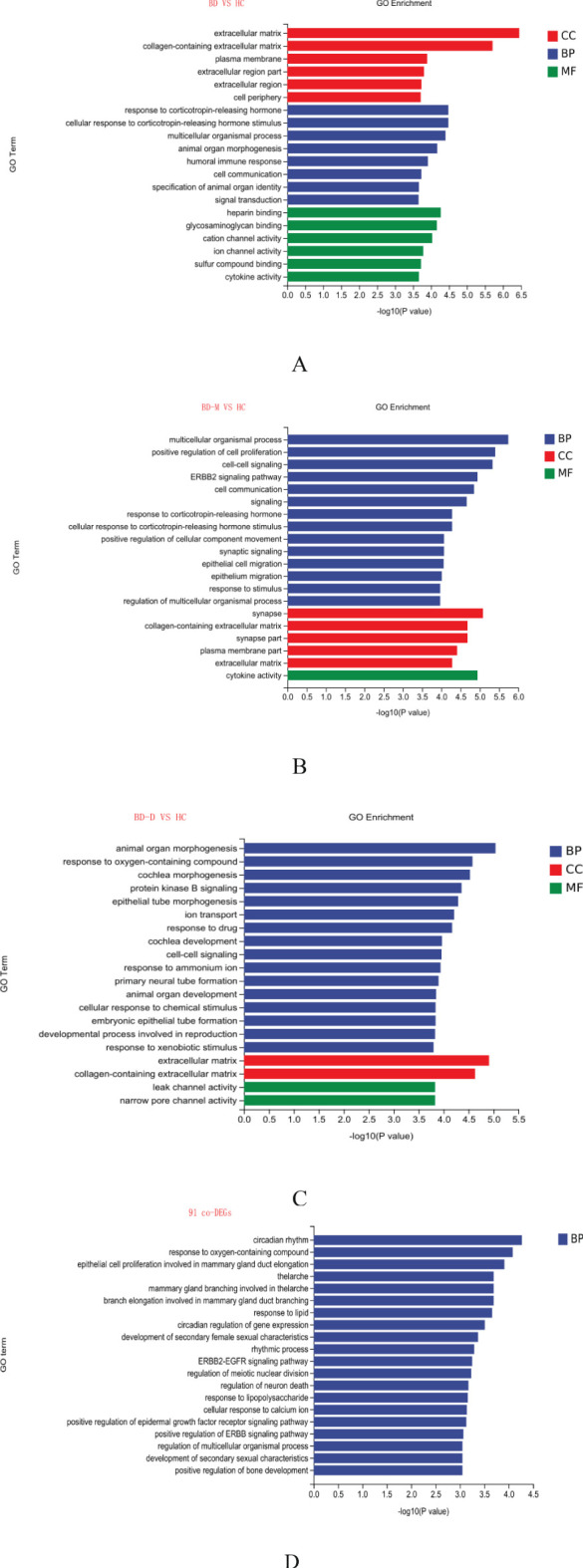
Gene Ontology (GO) of DEGs in BD vs. HC, BD-M vs. HC, BD-D vs. HC, and the 91 co-DEGs **(A-D)** (BP, represented in blue), (CC, in red), and (MF, in green).

GO Enrichment Analysis analysis categorized enriched GO terms into three main domains: biological process (BP),cellular component (CC), and molecular function (MF). The top 20 significantly enriched terms across these categories are presented (**Figures 2A–D**). For the broader BD vs. HC analysis, extracellular matrix components in CC suggest structural or signaling changes; the BP domain indicates stress response pathways related to corticotropin-releasing hormone; MF highlights heparin binding, affecting growth factor interactions or coagulation (**Figure 2A**).

In the comparison of BD-M vs. HC, the multicellular organismal process was identified as a top enriched term in BP, pointing toward changes in organism-level processes. For CC, the response to synapse underscored the potential impact on synaptic function. Cytokine activity was a prominent enriched term in MF, indicating an involvement in immune signaling (**Figure 2B**). Notable enriched terms for DEGs in BD-D vs. HC, Notable enriched terms for DEGs in BD-D vs. HC included animal organ morphogenesis in BP, extracellular matrix in CC, and leak channel activity in MF (**Figure 2C**). Among the 91 co-DEGs, circadian rhythm and response to oxygen-containing compounds were significant in BP (**Figure 2D**), highlighting potential disruptions in daily physiological rhythms and oxidative stress responses, respectively. No single GO term was enriched across all comparisons, suggesting distinct biological pathways in bipolar disorder and for each subtype.

### 3.4 KEGG enrichment analysis

**Figure 3:**
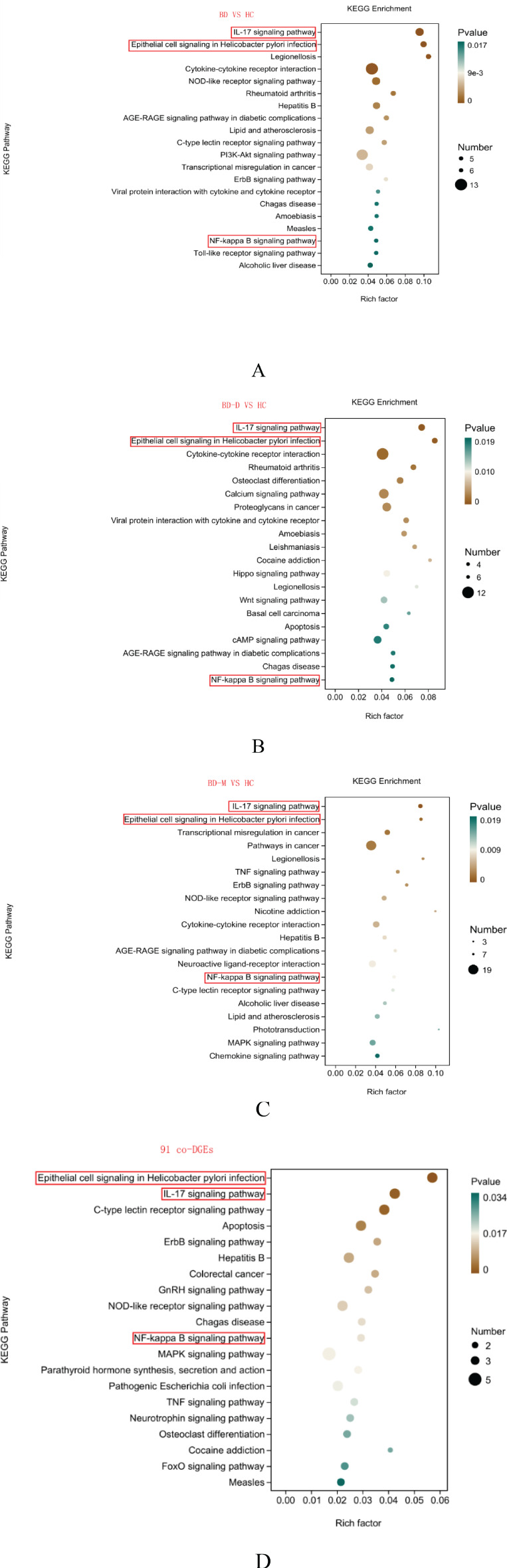
Enriched KEGG pathways in difference group BD vs. HC, BD-M vs. HC, BD-D vs. HC, and among the 91 co-DEGs **(A-D)**. The x-axis shows the enrichment factor for DEGs in pathways, while the y-axis lists significantly enriched pathways The color gradient represents P-values, with darker shades indicating lower values.

The KEGG enrichment analysis was conducted on the DEGs identified in the comparisons of BD vs. HC, BD-M vs. HC, BD-D vs. HC, and among the 91 co-DEGs, to delineate the functional pathways these genes are involved in. This analysis highlighted several specific pathways; notably, the IL-17 signaling pathway, epithelial cell signaling in Helicobacter pylori infection, and NF-kappa B signaling pathway emerged as common enriched pathways across all four groups (**Figures 3A–D**). The dot size on the plot reflects the number of DEGs in each pathway, with larger dots indicating more DEGs. A higher enrichment factor shows greater DEG enrichment, suggesting the pathway’s relevance to bipolar disorder.

### 3.5 Protein-protein interaction network analysis

**Figure 4:**
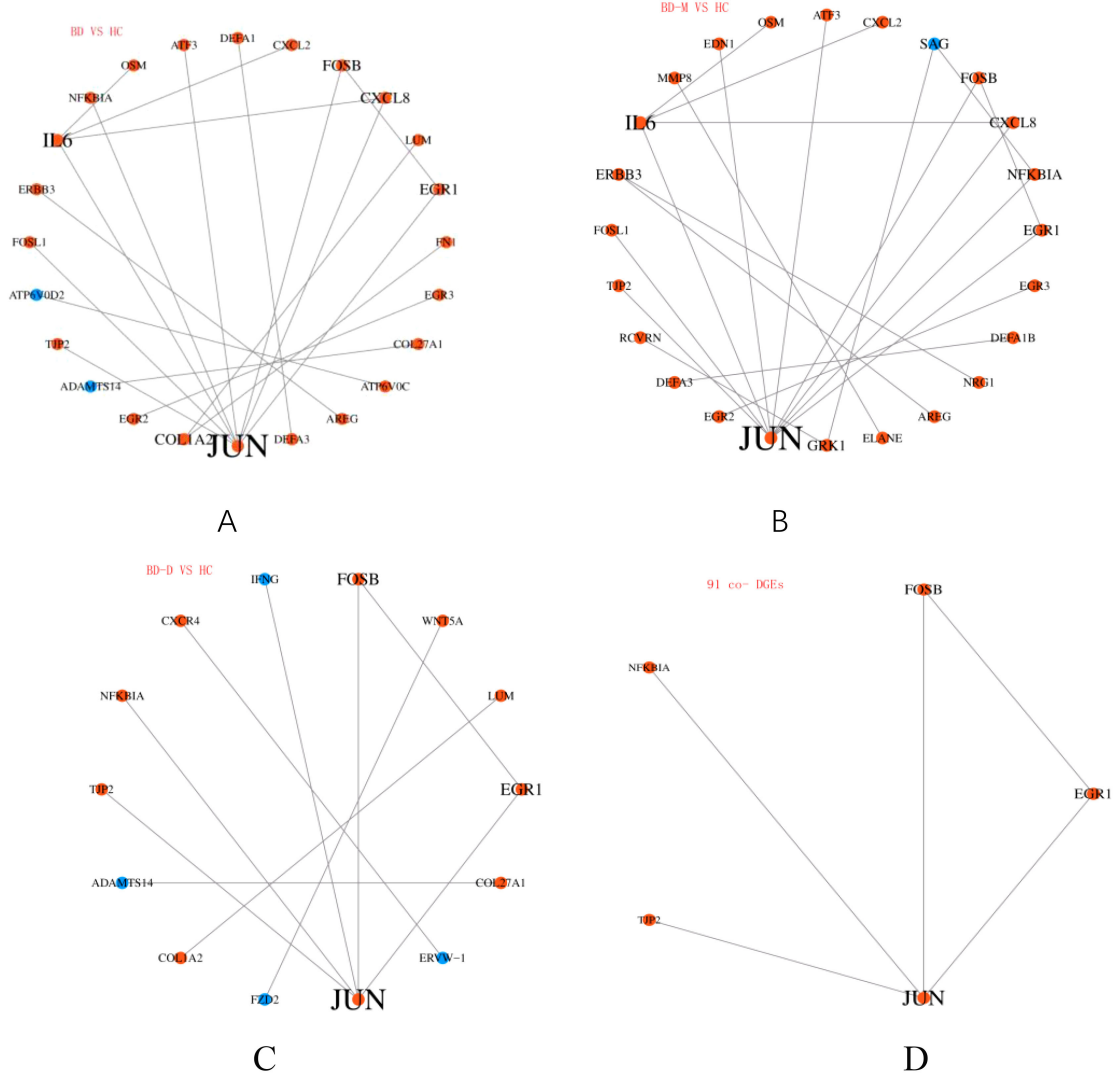
Protein-Protein Interaction (PPI) Network analysis For the DEGs in the BD vs. HC,BD-M vs. HC, BD-D vs. HC, and among the 91 co-DEGs **(A-D)**.

For the DEGs in the BD vs. HC comparison, the PPI network highlighted *JUN, FOSB, NFKBIA, IL-6*, and *CXCL8* as central nodes, suggesting these proteins may have pivotal roles in the pathophysiology of the depressed phase of BD (**Figure 4A**). These proteins, particularly due to their central positions within the network, could serve as potential biomarkers for BD. In the BD-M vs. HC comparison, the PPI network expanded to include *JUN, FOSB, FOSL1, NFKBIA, IL-6, and CXCL8* indicating their significant involvement in the manic phase of BD (**Figure 4B**). Similarly, for the broader BD-D vs. HC comparison, proteins such as *JUN, FOSB, and NFKBIA* were identified as playing crucial roles in BD, underscoring the complex interplay of signaling pathways and inflammatory processes in the disorder (**Figure 4C**).Among the 91 co-DEGs, *JUN and FOSB* emerged as core components of the network (**Figure 4D**), reinforcing their potential significance across different phases of BD and suggesting a consistent role in the disorder’s molecular basis.

### 3.6 IL-17 signaling pathway

**Figure 5:**
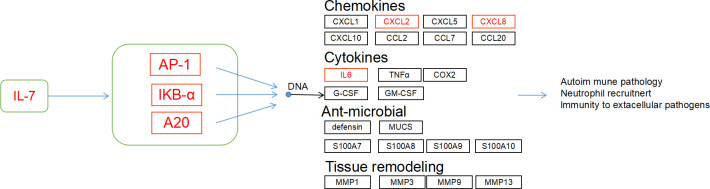
IL-17 signaling pathway was enriched in the BD vs. HC group. Red indicated the up-regulated transcript.

Focusing on the results of KEGG enrichment, core genes in the PPI analysis, and particularly the immune-related IL-17 pathway-which includes critical components such as AP-1 transcription factors (*Jun, Fosb, Fosl1), NFKBIA, TNFAIP3* (Zinc lipoprotein A20), and their downstream effectors like chemokines and cytokines*(CXCL2, CXCL8, IL-17* and *IL-6*). we identified significant gene expression upregulation in the IL-17 signaling pathway in BD, BD-M, BD-D compared to HC (**Figure 5**). Different result analyses have emphasized the relevance of the IL-17 pathway in BD and its subtypes, suggesting that the IL-17 signaling pathway may play a potential role in the pathogenesis of in BD and its subtypes.

### 3.7 PCR validation

**Figure 6:**
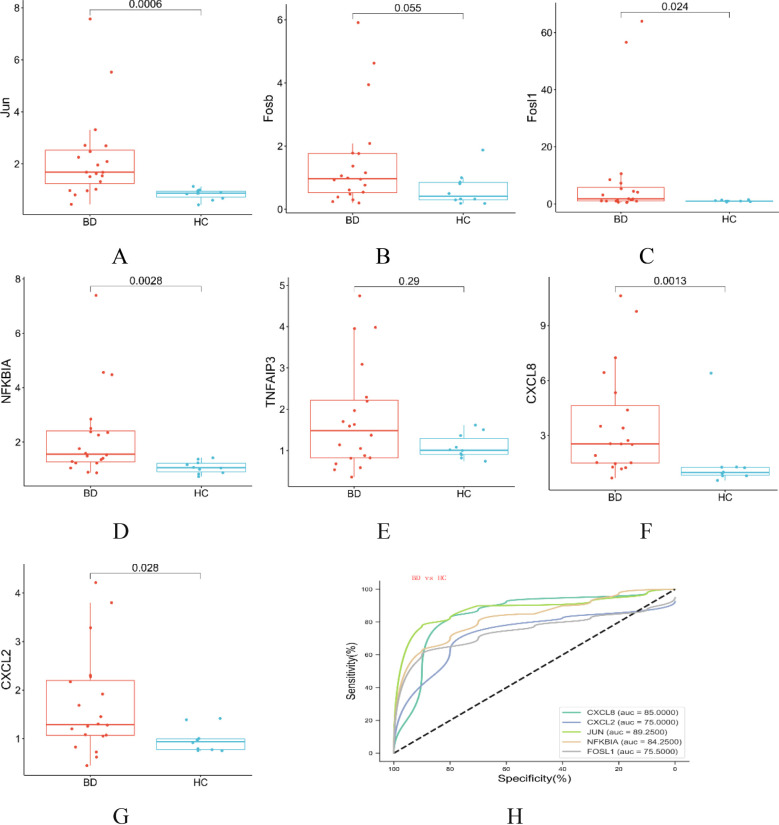
**(A-G)** Indicates the mRNA gene expression levels of the selected key genes (*Jun, Fosb, Fosl1, NFKBIA, TNFAIP3, CXCL8*, and *CXCL2*) validated by qPCR. **(H)** ROC curve analysis of qPCR results for *Jun, Fosl1, NFKBIA, CXCL8*, and *CXCL2.* (BD; n=20(BD-D n=10, BD-M n=10), HC; n=10; mean ± s.e., Red indicate high, while blue represent the low).

RT-qPCR experiments validated several key genes in the IL-17 signaling pathway, including *Jun*, *Fosb*, *Fosl1*, *NFKBIA*, *TNFAIP3*, *CXCL2* and *CXCL8*. The results indicated that the expression of these genes was significantly upregulated in BD, but only *Jun*, *Fosl1*, *NFKBIA*, *CXCL2* and *CXCL8* showed statistical significance (all *P* < 0.05) (**Figures 6A–G**).

Moreover, ROC curve analysis was performed, revealing significant diagnostic potential for several biomarkers: *Jun* (P < 0.001, AUC = 0.892, 95% CI [0.775, 1.000]), *NFKBIA* (P = 0.003, AUC = 0.843, 95% CI [0.704, 0.981]), *Fosl1* (P = 0.025, AUC = 0.755, 95% CI [0.583, 0.927]), *CXCL2* (P = 0.028, AUC = 0.75, 95% CI [0.569, 0.931]), and *CXCL8* (P = 0.002, AUC = 0.85, 95% CI [0.685, 1.000]), indicating their effectiveness in distinguishing between BD patients and healthy controls (**Figure 6H**).

### 3.8 Cytokine measurement via ELISA

**Figure 7:**
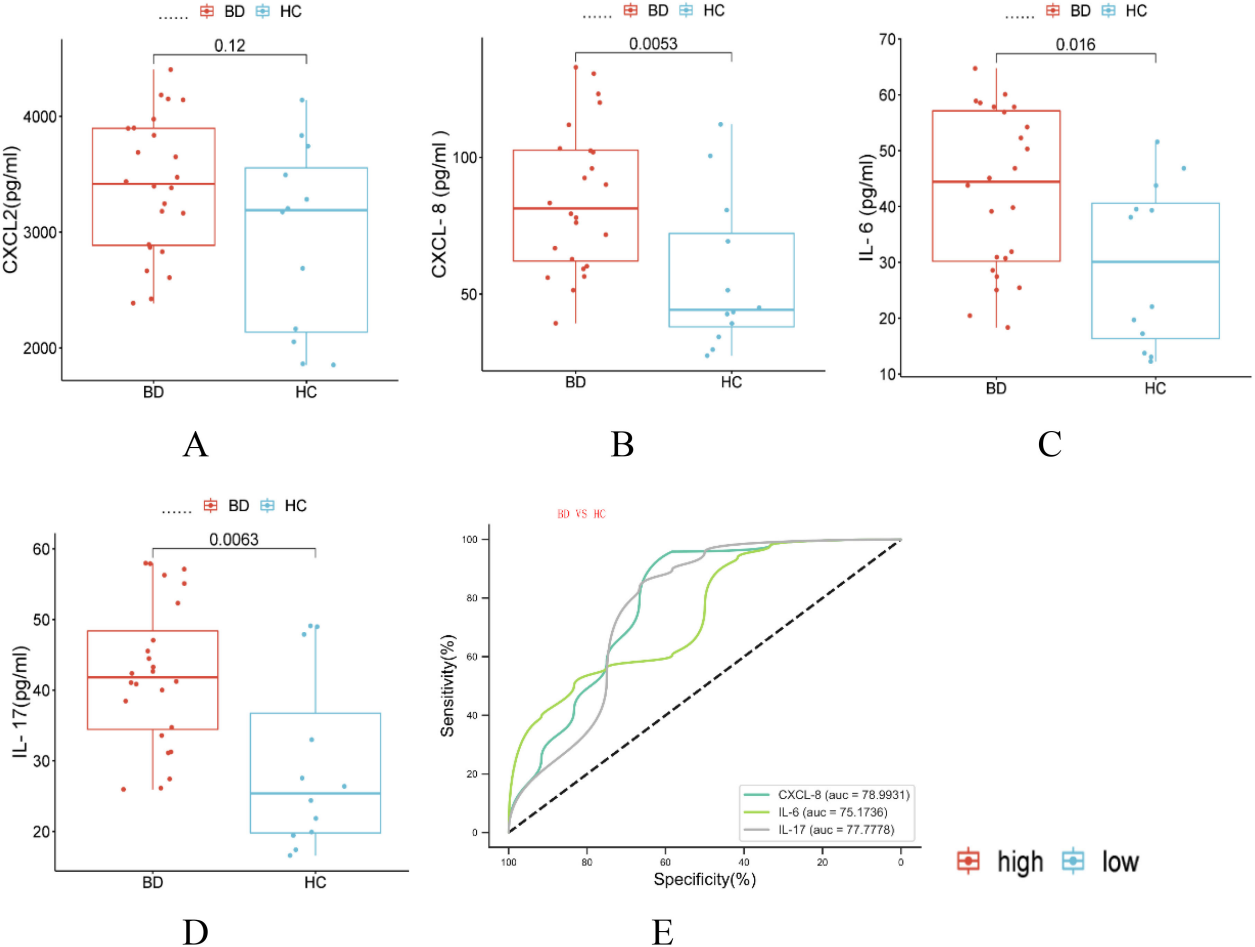
BD influences the expression Inflammatory cytokine expression. **(A-D)** Expression levels of cytokines CXCL2, CXCL8/IL-8, IL-6, and IL-17 in serum samples from the study participants were quantified using ELISA. **(E)** ROC curve analysis was conducted on CXCL2, CXCL8/IL-8, IL-6, and IL-17 expressed proteins to evaluate their diagnostic performance (BD; n=24(BD-D n=12, BD-M n=12), HC; n=12; mean ± s.e., Red indicate high, while blue represent the low).

Expression levels of cytokines CXCL2, CXCL8/IL-8, IL-6, and IL-17 in serum samples from the study participants (BD-D, BD-M, HC; n=12 each)) were quantified using ELISA. The results indicated an increase in CXCL2 expression among BD patients, but this difference was not statistically significant. In contrast, levels of CXCL8/IL-8, IL-6, and IL-17 were significantly elevated in the BD group compared to HC (all P < 0.05, **Figures 7A–D**).

ROC curve analysis was conducted on these differentially expressed proteins to evaluate their diagnostic performance. The analysis revealed that CXCL8 and IL-17 demonstrated notable diagnostic potential with AUC values of 0.79 (P=0.005, 95% CI: 0.614-0.965) and 0.778 (P=0.007, 95% CI: 0.59-0.965), respectively. IL-6 also showed significant predictive ability with an AUC of 0.752 (P=0.015, 95% CI: 0.584-0.919). Although CXCL2’s AUC of 0.663 (P=0.115, 95% CI: 0.663-0.857) suggested a trend toward diagnostic relevance, it did not reach statistical significance (**Figure 7F**).

### 3.9 Immune infiltration analysis

**Figure 8:**
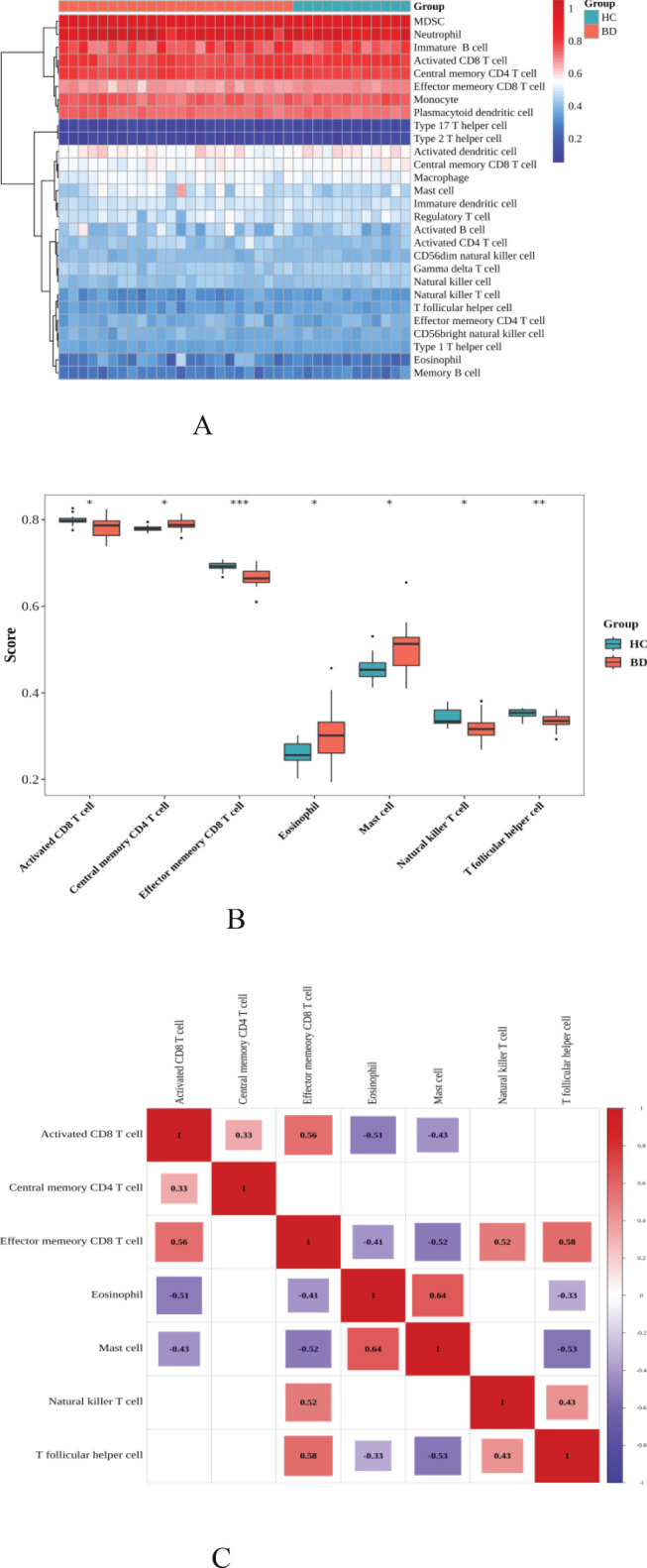
Utilizing Gene Set Variation Analysis (GSVA). **(A)** Heat maps are generated between immune cells from patients with BD vs HC; **(B)** Significant differences in immune cell infiltration between the BD and HC groups; **(C)** Correlation analysis of immune cells in the BD groups; the redder the color, the more representative of both the stronger the positive correlation; the more purple the color, the more representative of both the stronger the negative correlation) (BD; n=24(BD-D n=12, BD-M n=12), HC; n=12mean ± s.e., * indicated p < 0.05; ** indicated p < 0.01, *** indicated p < 0.001).

Utilizing Gene Set Variation Analysis (GSVA), we investigated the infiltration levels of 28 immune cell types in samples from individuals with BD and HC. The generated heatmap illustrated the relationships between various immune cells across all samples (**Figure 8A**). This analysis revealed significant differences in immune cell infiltration between the BD and HC groups. Specifically, central memory CD4+T cells (p=0.010), eosinophils (p=0.038), and mast cells (p=0.029) exhibited increased infiltration in the BD group. Additionally, natural killer T cells (p=0.015), T follicular helper cells (p=0.003), and activated CD8+T cells (p=0.038) were also significantly reduced in BD samples. Conversely, effector memory CD8+T cells showed a significant reduction in the BD group (p<0.001) (**Figure 8B**).

Notable correlations between immune cell types were observed: a strong positive correlation was found between effector memory CD8+T cells and activated CD8+T cells (r = 0.56), as well as between effector memory CD8+T cells and T follicular helper cells (r = 0.58). Eosinophils exhibited a positive correlation with mast cells (r = 0.645), while mast cells demonstrated negative correlations with T follicular helper cells (r = -0.53) and effector memory CD8+T cells (r = -0.52), highlighting intricate relationships within the immune landscape of BD (**Figure 8C**).

## 4 Discussion

In this study, we comprehensively analyzed changes in key genes in the IL-17 signaling pathway related to BD from a transcriptomic perspective. Utilizing microarray technology, we analyzed global mRNA expression in PBMC of BD patients compared to healthy controls, identifying 91 differentially expressed genes (DEGs) across various BD states-manic, depressive, and combined-against healthy controls. Transcriptome sequencing indicates a significant upregulation of genes in the IL-17 signaling pathway, such as (*Jun*, *Fosb*, *Fosl1*, *TNFAIP3*, *NFKBIA*, *CXCL2*, *CXCL8*, *IL-6* and IL-17) in BD patients. Some key genes were validated by qRT-PCR, and some cytokines were detected by ELISA, which showed significantly higher levels of CXCL8/IL-8, IL-6, and IL-17 proteins in BD patients. The study also revealed significant immune cell infiltration differences in BD, notably in central memory CD4+T cells, eosinophils, and mast cells. These findings underscore the crucial role of the IL-17 signaling pathway in BD’s immunoinflammatory response, and advancing our understanding of BD’s molecular mechanisms and the importance of the immune system in its pathology.

The identification of common pathways, especially the IL-17 signaling pathway, suggests an immunological aspect in BD and its subtypes(BD-D and BD-M), linking inflammation to psychiatric conditions. The NF-kappa B pathway’s enrichment indicates BD may involve altered immune response or chronic inflammation. The consistent enrichment of specific pathways across BD states highlights their significance in the disorder’s biology, necessitating further studies to clarify their roles.

The IL-17 signaling pathway plays a pivotal role in mediating biological effects through the regulation of key intracellular signaling molecules, including NF-κB activator 1 (ACT1), tumor necrosis factor receptor-associated factor 6 (TRAF6), NF-κB, and AP1 components such as JUN, FOSB, and TNFAIP3 (A20) (24–27). This pathway is crucial in activating inflammatory transcription factors, facilitating gene expression via NF-κB, and triggering the MAPK pathway (28). Emerging proteomic analyses indicate that the IL-17 signaling pathway is a crucial biological pathway shared by schizophrenia, bipolar disorder, and major depressive disorder (29). Moreover, previous research has identified the IL-17 signaling pathway as a shared enriched immunological pathway in schizophrenia and bipolar disorder, alongside their comorbidities, such as Type 2 Diabetes Mellitus (T2DM) (30). IL-17-dependent activation of iNOS (inducible nitric oxide synthase) is essential for microglial activation, promoting endothelial cell growth and accelerated vascular leakage and leukostasis via IL-6, producing a neuroinflammatory response (31). In our study, we compared BD samples to HC and found that nine factors associated with the IL-17 pathway (*Jun*, *Fosb*, *Fosl1*, *TNFAIP3*, *NFKBIA*, *CXCL2*, *CXCL8 IL-6* and *IL-17*) were upregulated in the transcriptome sequencing of bipolar disorder, and some of the upregulated genes were further validated by qPCR. ROC analysis underscored the diagnostic efficacy of these five gene expressions in BD and its subtypes, suggesting their potential utility in distinguishing BD patients from HC. These findings highlight the potential role of the IL-17 pathway in the pathogenesis of bipolar disorder, which is consistent with broader research linking this pathway to various psychiatric conditions. The increased levels of specific pathway components in BD patients improve our understanding of the disease.

IL-17 mediates inflammatory reactions in human nucleus pulposus cells by activating the p38/c-FOS and JNK/c-JUN pathways, both of which depend on AP-1 (32); AP-1 (activator protein-1) is a group of dimeric transcription factors that includes the subunits JUN, FOS, ATF (activating transcription factor), and MAF (musculoaponeurotic fibrosarcoma) subunits. The expression of various AP-1 dimers depends on the cell type and developmental stage. Additionally, AP-1 activity can display either pro-inflammatory or anti-inflammatory characteristics, depending on the cellular environment (33, 34). Alterations in AP-1 signaling within lymphocytes may contribute to the pathogenesis of BD or mirror the impact of antidepressant treatments (35). Our study finds that patients with bipolar disorder have higher levels of AP-1 transcription factor subunits *(Jun, Fosb, Fosl1)*. This suggests that AP-1 activation may play a role in modulating the immune-inflammatory response and neurogenesis in BD. AP-1’s involvement in these processes highlights its significance in the mechanisms of bipolar disorder. It provides insights into the complex interplay between genetic regulation, immune response, and neural development in this condition.

IL-6 and IL-17 are important pro-inflammatory cytokines, while CXCL2 and CXCL8/IL-8 function as chemokines that are pivotal to the immune-inflammatory response. Research indicates that BD has distinctive patterns of IL-6 gene expression. It also shows elevated levels of IL-6 secretion (12, 36). A comprehensive meta-analysis of 13 studies involving 1,221 patients with BD and 663 controls found that BD patients had significantly higher levels of interleukin-8 compared to the controls. Additionally, it noted an association between a longer duration of illness and lower levels of interleukin-8 (37). Moreover, increased interleukin-17 levels have been linked to suicidal behaviors in individuals with BD (38). Research indicates that IL-6 and CXCL2 levels are significantly increased in offspring at risk for mood disorders when compared to healthy counterparts, highlighting the role of genetic and environmental factors in inflammatory gene expression (39).In our study, IL-6, IL-17, and IL-8 levels were notably higher in the BD group than in healthy controls. ROC analysis confirmed that these cytokines are useful for diagnosing BD. This highlights their potential to distinguish BD patients from healthy individuals. Our findings align with prior research, reinforcing the critical role these cytokines may play in BD pathogenesis. It’s important to note the complex relationship between mRNA and protein levels for inflammatory cytokines, where correlations are not always straightforward. For example, although the mRNA expression level of CXCL2 was significantly higher in the BD group, the protein expression level of CXCL2 did not show a significant difference. This discrepancy underscores the complex regulatory mechanisms that control cytokine expression and action. It suggests that post-transcriptional and post-translational modifications, along with other regulatory processes, may affect the protein levels observed in BD.

IL-17 expression is often associated with a specific subset of CD4+ T cells and CD8+ T cells (40), CD4+T cells and CD8+T cells under particular conditions acquire the expression of Inflammatory factors (41), To delve deeper into the involvement of immune cells in BD, we utilized the CIBERSORT deconvolution method to analyze gene expression differences between BD patients and healthy controls. The analysis showed that individuals with BD had a significant increase in central memory CD4+ T cells, eosinophils, and mast cells compared to the control group. These findings are compliant with existing basic research and shed light shedding light on the immune mechanisms at play in BD. For instance, mast cells, known to accumulate at inflammation sites and release pro-inflammatory mediators, have been observed to decrease in circulation following pharmacological treatment in BD patients, underscoring their role in the disorder’s immune-inflammatory response. Similarly, natural killer (NK) cells were found in reduced numbers in BD patients, which may lead to the excessive activation of inflammatory cells (42). Single-cell immune analysis shows that treatment with quetiapine and sodium valproate for patients with bipolar disorder can trigger widespread anti-inflammatory effects. After treatment, the levels of pro-inflammatory mast cells and eosinophils decrease, which might help reduce inflammation by blocking immune signaling pathways mediated by myeloid cells, and, as a result, improve clinical symptoms (13). Moreover, our study examined correlations among 28 immune cell types, revealing positive associations between effector memory CD8+ T cells, activated CD8+ T cells, and T follicular helper cells in BD patients. Conversely, mast cells showed negative correlations with follicular helper T cells and effector memory CD8+T cells, highlighting complex interactions between different immune cell types in BD. These correlations suggest both synergistic and antagonistic relationships among immune cells, influencing the course of the disorder. Current basic research into the correlation analysis among various immune cell types in BD has not been extensively validated on a large scale, rendering our predictive findings particularly valuable. These findings serve as a foundational reference for understanding the complex dynamics of immune cells in bipolar disorder (BD) and open avenues for future research into these relationships.

This study represents a comprehensive and innovative exploration into the transcriptomic landscape of BD, focusing on changes in inflammatory components in the IL-17 signaling pathway.

This finding aligns with emerging research that links inflammatory processes to psychiatric conditions, thus advancing our understanding of BD’s molecular mechanisms and emphasizing the immune system’s importance in its pathology. The use of comprehensive bioinformatics analyses, including GO, KEGG pathway enrichment, and Protein-Protein Interaction (PPI) analysis, alongside Gene Set Variation Analysis (GSVA) for immune cell infiltration, provides a holistic view of the disorder’s underlying biology, highlighting the interaction of genetic factors and the immune response.

The results of this study are significant because they indicate the presence of a potential immunological inflammatory component in BD, which may provide new ideas for the development of novel treatment strategies for patients with BD. This is consistent with increasing evidence that psychiatric disorders, including bipolar disorder, may involve changes in immune responses or chronic inflammation.

Several limitations of our study need discussion. Firstly, the relatively small sample size could have diminished the statistical power necessary for robust comparison of gene expression between BD patients and healthy controls. Future studies with larger sample sizes are warranted to validate our findings. Secondly, discrepancies were observed in the direction of gene expression alterations between the microarray analysis and the subsequent qRT-PCR validation. This could be attributed to the use of different samples for validation purposes, as well as the variability in disorder stages and treatment regimens among the patients. Thirdly, the prediction of immune cell involvement was based on the CIBERSORT deconvolution method. While this algorithm has been extensively applied and validated in tumor prognostic gene studies, its application in psychiatric disorders remains relatively unexplored. Consequently, further experimental validation in psychiatric disorders is essential to confirm these preliminary findings. Fourth, This study implemented a two-week drug washout period, but we recognize that a two-week clearance period may not be sufficient to completely eliminate the drug’s impact on the study results. Future research will consider a longer withdrawal period. Fifth, although differential gene analysis strongly suggests the role of IL-6 and IL-17 signaling in the pathogenesis of the disease, our study lacks qPCR validation of these targets at the mRNA level; future research should prioritize conducting parallel transcriptomic-proteomic analyses.

## 5 Conclusion

This study offers significant insights into the role of IL-17 signaling in modulating the immune response in BD, establishing a robust association between this pathway and the condition. Furthermore, the study revealed significant variations in immune cell infiltration in BD, which contributes to a better understanding of the disorder’s immunoinflammatory mechanisms. These findings emphasize the immune system’s role in BD that suggest targeting the IL-17 signaling pathway could offer novel therapeutic avenues.

## Data Availability

The original contributions presented in the study are publicly available. This data can be found here: NCBI BioProject, accession PRJNA1050039.
