# Supplementary material for: The alteration of IL-17 signaling pathway in bipolar disorder: a preliminary study with transcriptomic perspective
Source: Front Psychiatry. 2025 Jun 5;16:1539038. doi: 10.3389/fpsyt.2025.1539038 (PMC12176804; doi:10.3389/fpsyt.2025.1539038)
Supplement: Supplementary file 1 [file DataSheet1.pdf]

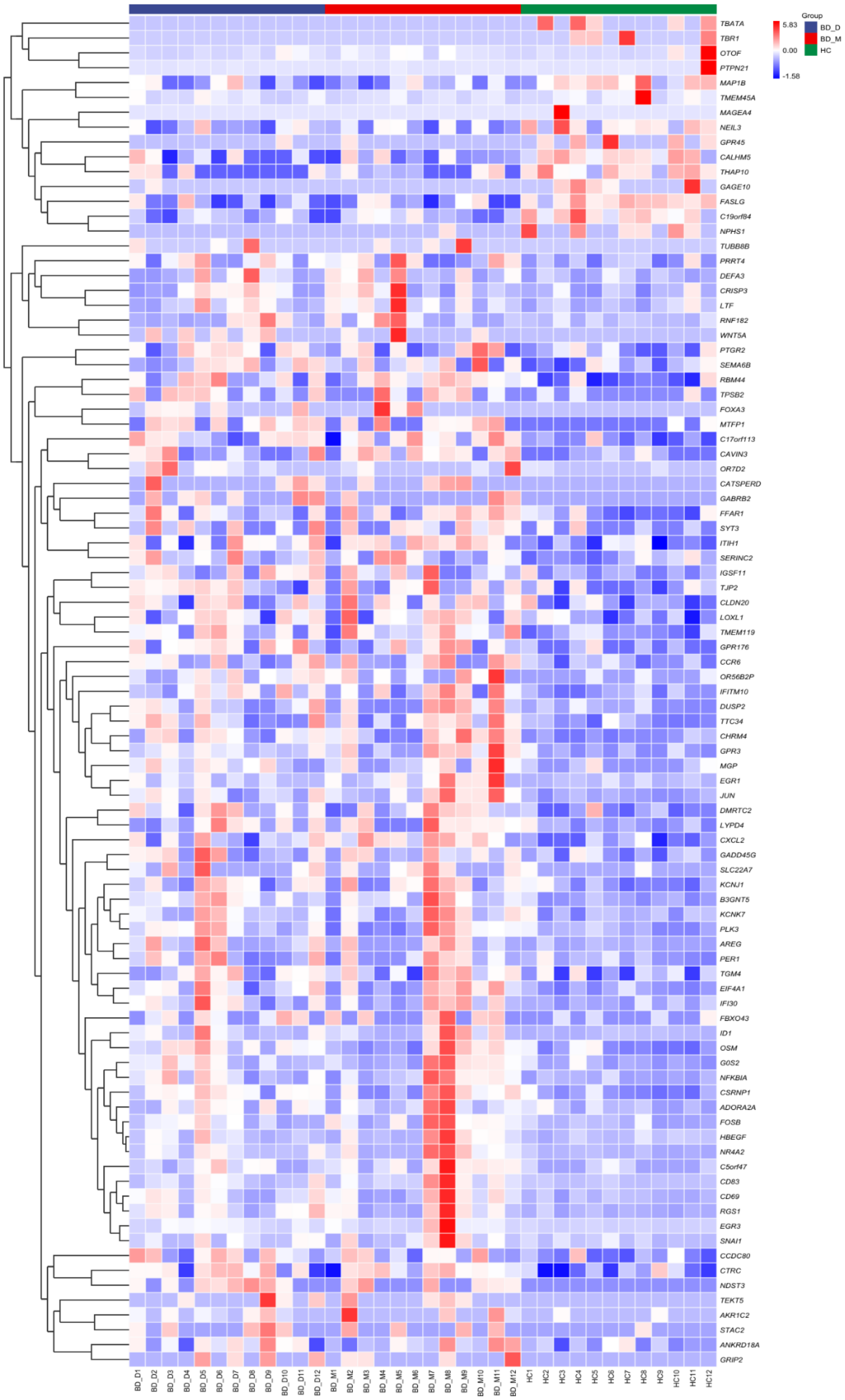

Group
BD_D
BD_M
HC
5.83
0.00
-1.58
TBATA
TBR1
OTOF
PTPN21
MAP1B
TMEM45A
MAGEA4
NEIL3
GPR45
CALHM5
THAP10
GAGE10
FASLG
C19orf84
NPHS1
TUBB8B
PRRT4
DEFA3
CRISP3
LTF
RNF182
WNT5A
PTGR2
SEMA6B
RBM44
TPSB2
FOXA3
MTFP1
C17orf113
CAVIN3
OR7D2
CATSPERD
GABRB2
FFAR1
SYT3
ITIH1
SERINC2
IGSF11
TJP2
CLDN20
LOXL1
TMEM119
GPR176
CCR6
OR56B2P
IFITM10
DUSP2
TTC34
CHRM4
GPR3
MGP
EGR1
JUN
DMRTC2
LYPD4
CXCL2
GADD45G
SLC22A7
KCNJ1
B3GNT5
KCNK7
PLK3
AREG
PER1
TGM4
EIF4A1
IFI30
FBXO43
ID1
OSM
G0S2
NFKBIA
CSRNP1
ADORA2A
FOSB
HBEGF
NR4A2
C5orf47
CD83
CD69
RGS1
EGR3
SNAI1
CCDC80
CTRC
NDST3
TEKT5
AKR1C2
STAC2
ANKRD18A
GRIP2
BD_D1
BD_D2
BD_D3
BD_D4
BD_D5
BD_D6
BD_D7
BD_D8
BD_D9
BD_D10
BD_D11
BD_D12
BD_M1
BD_M2
BD_M3
BD_M4
BD_M5
BD_M6
BD_M7
BD_M8
BD_M9
BD_M10
BD_M11
BD_M12
HC1
HC2
HC3
HC4
HC5
HC6
HC7
HC8
HC9
HC10
HC11
HC12

S1

S1. Heat map for 91 DEGs in buffy coat from patients with BD-D, BD-M vs HC. Each row represents a gene and each column represents a sample. Patients are classified as BD-M (blue),BD-D (red)or HC (green) as indicated at the bottom of the heat map. Red and blue indicate expression levels above and below the median, respectively. The dendrogram to the left of the matrix represents overall similarities in gene expression profiles.
